# Newly diagnosed glioblastoma IDHwt patients treated with radiation, nivolumab, and BMS-986205

**DOI:** 10.21203/rs.3.rs-7567487/v1

**Published:** 2025-09-25

**Authors:** Derek Wainwright, Rimas Lukas, Lijie Zhai, Kristen Lauing, Miri Kim, Taylor Koch, Manon Penco-Campillo, Prashant Bommi, Karan Dixit, Priya Kumthekar, Laura Sharp, Raymond Lezon, Danyelle Garcia, Adam Sonabend, Kathleen McCortney, Brandyn Castro, James Chandler, Vinai Gondi, Sean Grimm, Amy Heimberger, Katrina Dobinda, Hui Zhang, Sean Sachdev, Csaba Juhasz, C. David James, Jacob Allen, Craig Horbinski, Maciej Lesniak, Roger Stupp, Jason Miska

**Affiliations:** Loyola University Chicago Stritch School of Medicine; Northwestern University; Loyola University Chicago Stritch School of Medicine; Loyola University Chicago Stritch School of Medicine; Loyola University Chicago Stritch School of Medicine; Loyola University Chicago Stritch School of Medicine; Loyola University Chicago Stritch School of Medicine; Loyola University Chicago Stritch School of Medicine; Northwestern University Feinberg School of Medicine; Northwestern University; Northwestern University Feinberg School of Medicine; Northwestern University Feinberg School of Medicine; Northwestern University Feinberg School of Medicine; Northwestern University; Northwestern University; Northwestern University Feinberg School of Medicine; Northwestern University Feinberg School of Medicine; Northwestern University Feinberg School of Medicine; Northwestern University Feinberg School of Medicine; Northwestern University; Northwestern University Feinberg School of Medicine; Northwestern University; Northwestern University Feinberg School of Medicine; Wayne State; Feinberg School of Medicine, Northwestern University; University of Illinois Champagne-Urbana; Northwestern University; Northwestern University; Northwestern University; Northwestern University

**Keywords:** indoleamine 2,3 dioxygenase 1, IDO, PD-1, immunotherapy, immune checkpoint, glioma

## Abstract

This phase I trial evaluated the IDO1 enzyme inhibitor, BMS-986205, with radiation (RT) and nivolumab treatment in newly diagnosed patients with GBM IDHwt. Cohort A received RT + nivolumab with escalating BMS-986205 doses in MGMT unmethylated GBM patients. Cohort B received the highest dose of BMS-986205 with nivolumab and standard RT/temozolomide (TMZ) TMZ in MGMT methylated GBM patients. The treatments were found to be safe and tolerable. The median overall survival was 11.5 (95% CI: 3.71, 33.8) and 26.9 months (95% CI: 8.94-NR) while the 2-year survival rates were 33% (95% CI: 10.3%, 58.8%) and 60% (95% CI: 12.6%, 88.2%) for MGMT unmethylated and methylated GBM, respectively. Longer patient survival was associated with higher CD8^+^ T cell levels, higher microbial aryl-lactate levels, higher abundance of *Massilioclostridium coli*, *Dysosmobacter welbionis*, and *Phocaeicola plebeius* in the stool, a younger age, and a lack of gross total resection. (ClinicalTrials.gov: NCT04047706).

## INTRODUCTION

Glioblastoma (GBM; IDH wild-type) is a highly aggressive incurable primary central nervous system (CNS) tumor^1^. Standard of care (SOC) treatment consists of maximum surgical resection, radiation therapy (RT) with concomitant temozolomide chemotherapy (TMZ)^2^, followed by adjuvant TMZ and tumor treating fields (TTF)^3^. Tumors with an unmethylated O^6^-methylguanine-DNA methyltransferase (MGMT) gene promoter have an inferior prognosis and benefit less from TMZ^4^.

In contrast to the broad success of immunotherapy for treating cancer that arises outside of the CNS, immune checkpoint inhibitors have failed to improve outcome in randomized trials in newly diagnosed and recurrent glioma^5,6^. We hypothesized that this immunotherapeutic resistance may be overcome by targeting the pleiotropic upstream immunosuppressive factor, indoleamine 2,3-dioxygenase 1 (IDO; IDO1)^7^. IDO1 is an interferon-inducible rate-limiting enzyme that metabolizes the least abundant essential amino acid, *L*-tryptophan, into downstream, *L*-kynurenine^8,9^. Higher IDO1 expression in patient-resected GBM is inversely associated with overall survival (OS)^10,11^, and preclinical models with glioma cells knocked down for IDO1 expression show a significant survival advantage^12^. While pharmacologic IDO1 enzyme inhibitor treatment fails to improve survival as a monotherapy or dual therapy when combined with an anti-PD-1 mAb, the triple treatment of RT, PD-1 mAb, and an IDO1 enzyme inhibitor leads to therapeutic synergy and durable survival improvement among multiple preclinical brain tumor models^13^. RT initiates substantial cytoreductive tumor cell death and activates the cGAS/STING pathway subsequently inducing interferon signaling^14^. Since interferon signaling potently induces IDO1 expression and activity in glioblastoma^11^, we hypothesize that immunostimulatory effects of RT are hindered by IDO1-mediated immunosuppression.

We initiated a phase I clinical trial (NCT04047706) to evaluate safety and tolerability for the triple combination of radiotherapy, nivolumab (anti-PD1 antibody), BMS-986205 (IDO enzyme inhibitor) in patients with newly diagnosed GBM. The first cohort was for tumors with unmethylated *MGMT*, thus allowing omission of TMZ from the SOC regimen. In a subsequent second cohort, *MGMT* methylated tumors were treated with the investigational regimen + standard TMZ. Secondary objective included evaluation of efficacy including OS and progression-free survival (PFS) as well as extensive correlative studies.

## RESULTS

### Patient demographics, adverse events, response rates, and survival

#### Cohort A: MGMT promoter unmethylated GBM

**Figure 1:**
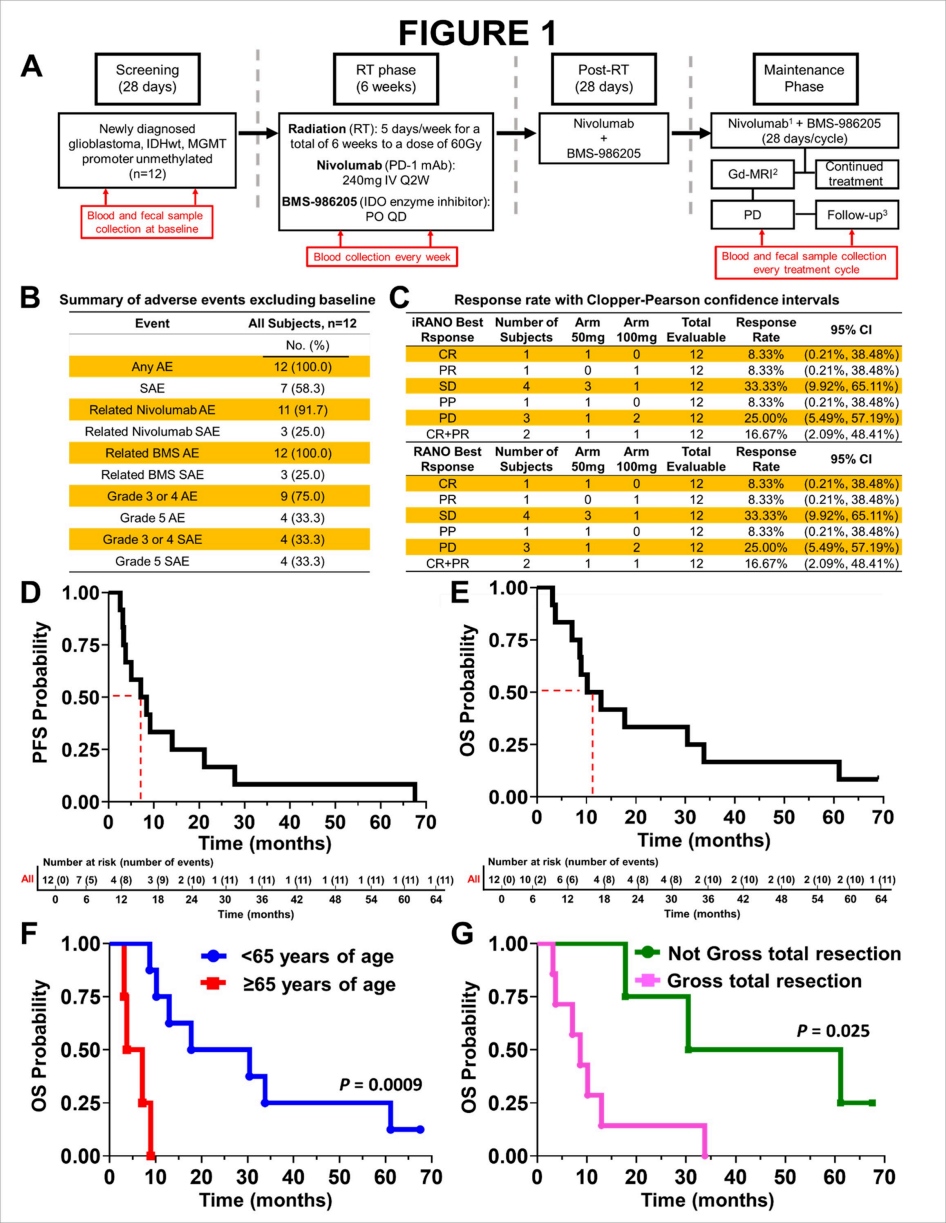
Trial overview, adverse events, response rates, and overall survival of cohort A GBM patients treated with radiation, nivolumab, and BMS-986205. **(A)** Study schema for the phase 1 clinical trial combining standard radiation (RT), nivolumab (anti-PD-1 mAb), and BMS-986205 (IDO1 enzyme inhibitor) for treating newly-diagnosed IDHwt MGMT promoter unmethylated patients with glioblastoma (listed on clinicaltrials.gov as NCT04047706). ^1^Beginning at Cycle 6 of the Maintenance Phase, nivolumab was given at 480mg Q4W IV. ^2^Gd-MRI occurred every 8 weeks during the Maintenance Phase beginning on Cycle 1 Day 1 (C1D1). ^3^Patients were contacted every 3 months either by clinic visit or by telephone to monitor survival. IV Q2W: intravenously once every 2 weeks; PO QD: orally once a day; PD: progressive disease. **(B)** Summary of adverse events reported for the 12 GBM patients (cohort A) in the study. **(C)** The iRANO and RANO response rate associated with the 12 patients in cohort A. **(D)** *Top panel*: Progression free survival (PFS) analysis of cohort A GBM patients. Dashed line indicates the median PFS; *Bottom panel*: number at risk during designated survival time. **(E)** *Top panel*: Kaplan-Meier curve for overall survival (OS) of the 12 GBM patients reported in the study. Dashed line indicates the median OS; *Bottom panel*: number at risk at designated survival time. Kaplan-Meier curve for overall survival (OS) as stratified by **(F)** age and **(G)** surgical resection type.

Twelve patients with newly diagnosed MGMT unmethylated GBM IDHwt (Cohort A) and 6 patients with MGMT methylated tumors were enrolled (Cohort B) (Fig. 1A). Demographic and clinical characteristics are summarized in Table 1. Median age at enrollment was 58 years (range 41–69 years). Fifty-eight percent were male and 42% female. All patients had a baseline KPS ≥70, with 5 individuals at 70–80, and the remaining 7 patients with KPS ≥90. The dose of the IDO enzyme inhibitor (IDOi) BMS-986205 was escalated from 50 mg (n=6) to 100 mg (n=6) daily. The first dosing cohort (50mg; n=6) was followed by a second (100mg; n=6) of BMS-986205 daily. A limited number of patients (n=2; longest surviving MGMT unmethylated patients) discontinued treatment due to loss of BMS-986205 availability. All patients who consented to autopsy at the time of death showed a diffuse spread of tumor throughout the brain including the brainstem as previously described and the treatment did not alter expected pattern of end stage disease progression^19^.

The therapeutic regimen was overall well tolerated with treatment emergent adverse events (TEAE) mostly related to radiation, TMZ, or the underlying disease and tumor progression (Fig. 1B; **Supplementary Table S1**). There are few serious AEs (SAEs) (**Supplementary Table S2, S3**). The treatment-related AEs (TRAEs) were predominantly lower grade (**Supplementary Table S4, S5**) with no significant difference observed between the IDO1 inhibitor dose cohorts (**Supplementary Table S6**). Methemoglobinemia was not observed in this study, in contrast to other clinical trials with BMS-986205, likely due to the lower doses (100 vs 200 mg) given^15^. Dose limiting toxicities (DLTs) as reflected by increased transaminases (grade 3) were observed in 2 and 3 patients at 50mg and 100mg levels of BMS-986205, respectively, while malaise was only observed in the 50mg arm (**Supplementary Table S7**). The 50mg daily schedule was established as the recommended phase 2 dose when used in conjunction with RT and nivolumab.

Concordance was observed between RANO and iRANO responses for individual patients. Response rate as defined according to either RANO or iRANO criteria was 8.33% complete responses (CR), 8.33% partial responses (PR), and 16.67% CR + PR. Stable disease (SD) was observed in 33.33% and progressive disease (PD) in 25% (Fig. 1C). The median PFS was 7.74 months (95% two-sided CI: 3.15, 21.2) (Fig. 1D). The median overall survival (mOS) was 11.5 months with the 6-month, 12-month, 24-month, 36-month and 48-month landmark survival probabilities at 83.3%, 50.0%, 33.3%, 16.7% and 16.7%, respectively (Fig. 1E, **Supplementary Table S8**). Longer OS was observed for patients <65 years of age compared to ≥65 years (Fig. 1F; *P*=0.0009) and those who did not receive a gross total resection as compared to individuals who underwent gross total resection (Fig. 1G; *P*=0.025). Multivariable Cox proportional model analysis confirmed that age was an independent and significant predictor of OS (**Supplementary Table S9**). The OS did not vary with differences in BMS-986205 dosage (**Supplementary Fig. S1**).

#### Cohort B: MGMT promoter methylated GBM

Six patients with MGMT promoter methylated GBM IDHwt were enrolled in Cohort B. Demographic and clinical baseline characteristics of patients in this cohort are listed in Table 1. Median patient age at enrollment was 64.5 years old (range 55–72 years). BMS-986205 was dosed 1 level below the established phase 2 dose of 50 mg daily (n=6 at 25mg daily) due to the addition of the TMZ. The dose was not escalated due to DLTs. Three patients opted to use TTF. The regimen was well tolerated with predominantly lower grade AEs (**Supplementary Tables S10, S11**) and limited SAEs (**Supplementary Tables S12, S13**). TRAEs were predominantly lower grade (**Supplementary Tables S14, S15**). Cerebral edema was the only dose-limiting toxicity (**Supplementary Table S16**). No response was observed according to either RANO or iRANO (**Supplementary Table S17**). SD was observed for 66.67% and 50% according to RANO and iRANO, respectively, with suspected pseudoprogression in 33% and 50% per RANO and iRANO (**Supplementary Table S17**). The mOS for the MGMT promoter methylated cohort was 26.9 months (**Supplementary Fig. S2**), while the 6-month, 12-month, 24-month and 36-month landmark survival probabilities were 100%, 80%, 60%, and 40%, respectively (**Supplementary Table S18**). Median PFS was 13.1 months (**Supplementary Fig. S3**). Since the larger group of individuals with MGMT unmethylated GBM were enrolled into the clinical trial prior to opening the enrollment to the smaller group of MGMT methylated patients, and because our expectation was that the MGMT unmethylated GBM patients would experience an accelerated clinical disease course, and that TMZ could potentially abrogate efficacy of the regimen, the following correlative measures focused on the analysis of patients in cohort A with MGMT unmethylated GBM.

### Metabolite analyses

**Figure 2:**
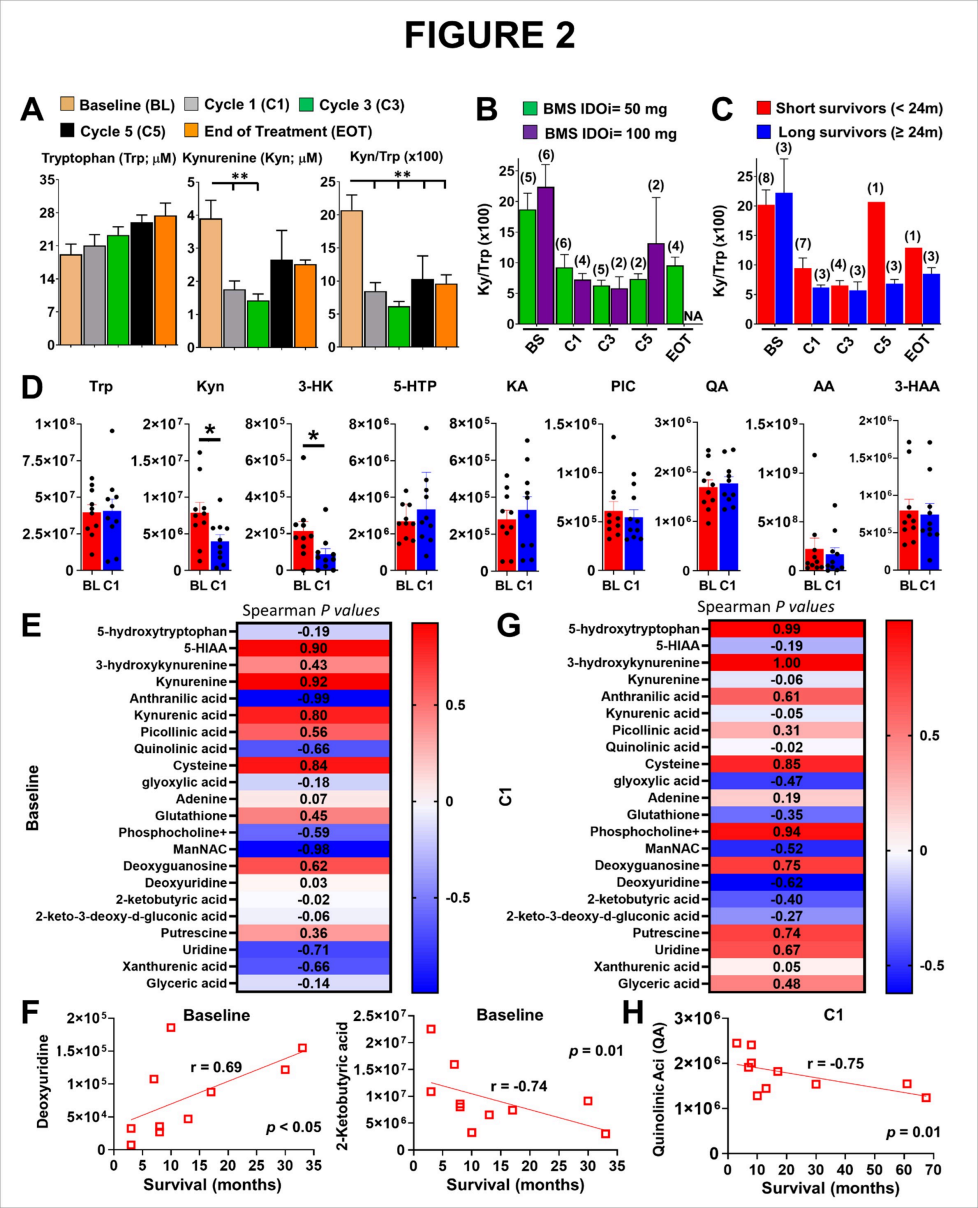
Peripheral blood metabolite analysis of cohort A. **(A)** The peripheral blood level of *L-*tryptophan (Trp) and *L-*kynurenine (Kyn) were measured by HPLC-MS/MS using serum samples collected at designated time points of the trial. BL: baseline (n=11); C1: cycle 1 day 1 of the maintenance phase (n=10); C3: cycle 3 day 1 of the maintenance phase (n=7); C5: cycle 5 day 1 of the maintenance phase (n=4); EOT: end of treatment of the maintenance phase (n=4). Comparison of Trp/Kyn ratios between **(B)** BMS-986205 dosing arms and **(C)** long- vs. short-term survivors. Samples size for each group is shown in paratheses. (**D)**Comparison of serum-derived major Trp-Kyn pathway metabolites between baseline (BL, n=10) and on-treatment (C1, n=10). **(E)** Non-parametric Spearman correlations assessing baseline (BL) serum metabolites vs. future survival time. Blue: negative correlation; red: positive correlation. **(F)** Two serum metabolites show a significant correlation with survival in the baseline sample analysis. **(G)** Non-parametric Spearman correlations assessing on-treatment (C1) serum metabolites vs. future survival time. Blue: negative correlation; red: positive correlation. **(H)** One serum metabolite, quinolinic acid, shows a significant correlation with survival in the on-treatment sample analysis. * *P*<0.05.

To evaluate the influence of RT + nivolumab + BMS-986205 on the IDO1-mediated *L*-tryptophan (Trp) → *L*-kynurenine (Kyn) pathway, we conducted mass spectrometry-based metabolic analysis on plasma samples. An early decrease in systemic Kyn levels and a sustained decrease of the Kyn/Trp ratio was observed throughout the RT and maintenance phases (Fig. 2A). There was no dose response difference between BMS-986205 and the Kyn/Trp ratio (Fig. 2B) and the Kyn/Trp ratio was not different between long- and short-term surviving patients (Fig. 2C). Except for Kyn and 3-hydroxykynurenine (3-HK), there were no significant changes for Kyn pathway metabolites including Trp, 5-hydroxytryptophan (5-HTP), kynurenic acid (KA), picolinic acid (PIC), quinolinic acid (QA), anthranilic acid (AA), or 3-hydroxy-anthranilic acid (3-HAA) between the baseline (BL) and cycle 1 of the maintenance phase (C1) (Fig. 2D, **Supplementary Fig. S4A, S4B**). We compared other metabolites between BL and C1 time points and selected those with significant differences for non-parametric Spearman correlation analysis with survival (Fig. 2E). Of baseline samples, deoxyuridine and 2-ketobutyric acid significantly correlated with OS (Fig. 2F). Similarly, Spearman correlation analysis of C1 samples (Fig. 2G) identified a significant correlation between quinolinic acid levels and OS (Fig. 2H). Pathway enrichment analysis identified taurine and hypotaurine metabolism, glutathione metabolism, and amino/nucleotide sugar metabolism as secondary but notable metabolic shifts after treatment as well (**Supplementary Fig. 4C**). These results confirm that IDO enzyme inhibitor treatment selectively decreases metabolites in the kynurenine pathway alongside broader metabolic reprogramming.

### Peripheral blood mononuclear cell analysis

**Figure 3:**
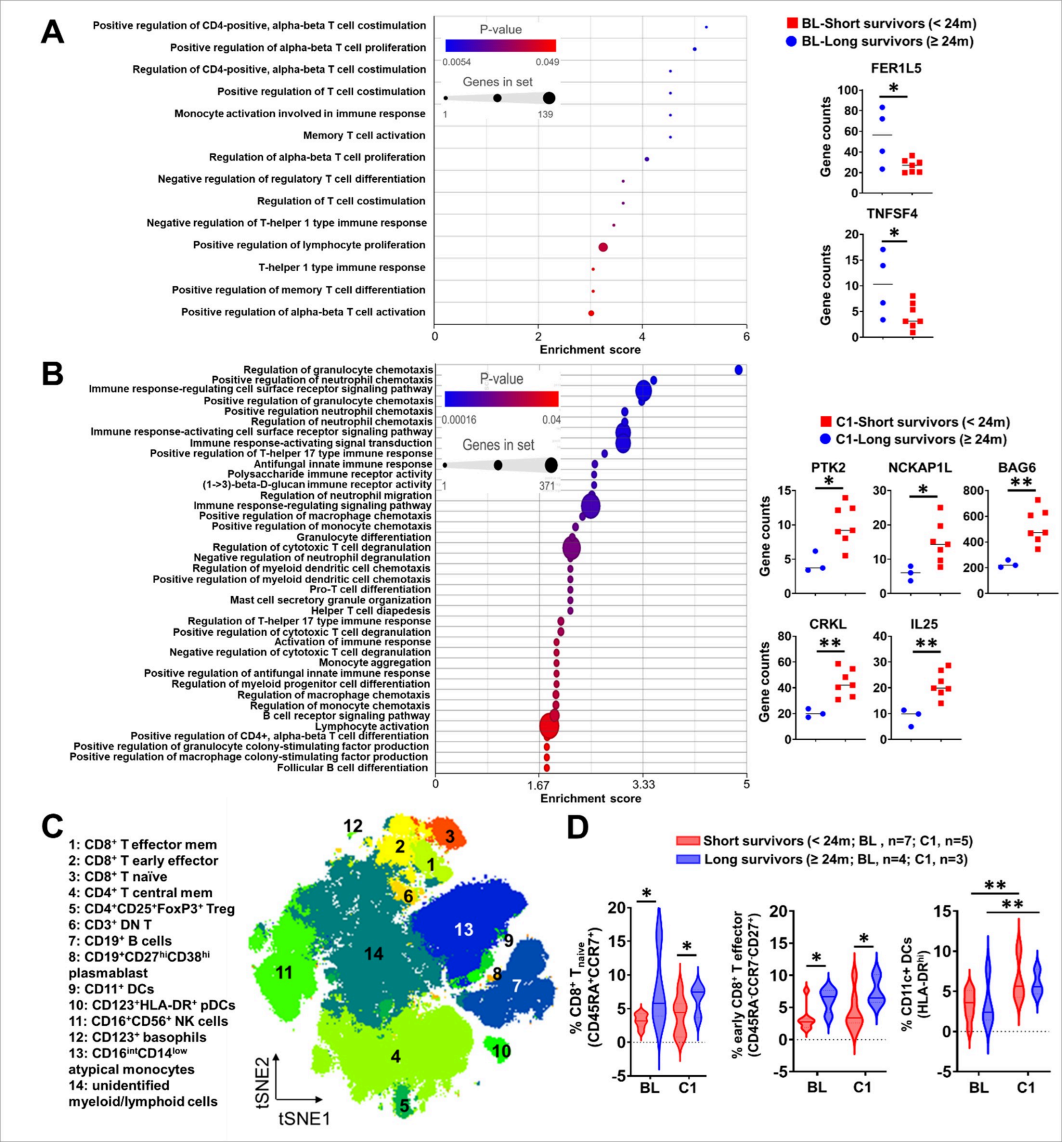
PBMC analysis of immune-related gene expression and immune cell analysis of longer- and shorter-surviving GBM patients in cohort A. RNA-Seq was performed on PBMC samples from enrolled patients. **(A)** Differential gene expression was compared between GBM patients that survived ≥24 months (Long Survival, n=4) and <24 months (Short Survival, n=8) from PBMCs isolated at baseline (BL). *Left panel*: significant immune-related GO pathways were identified by Gene Set Enrichment with *P*<0.05; *Right panel*: comparison of expression levels for immune-related GO genes that were highlighted during the Gene Set Enrichment analysis. **(B)** Differential gene expression was also compared between long- (n=3) and short-term (n=7) survival patient PBMCs isolated at the cycle 1 day 1 (C1) time point. *Left panel*: Significant immune-related GO pathways identified by Gene Set Enrichment with *P*<0.05; *Right panel*: comparison of expression levels for immune-related GO genes that were highlighted during the Gene Set Enrichment analysis. **(C)** t-SNE plot projection of immune cells in the peripheral blood via spectral flow cytometry analysis. **(D)** Comparison of different CD8^+^ T cell subtypes and dendritic cells (DCs) between baseline and C1 timepoints stratified by short-term vs. long-term survival. *P<0.05, **P<0.01.

Gene set enrichment analysis (GSEA) revealed that treatment with radiation, nivolumab, and BMS-986205 globally reprogrammed the immune system (**Supplementary Fig. S5A**) in peripheral blood mononuclear cells (PBMCs). When compared to baseline and C1 time points, treatment-induced gene changes included TRIM62 and XKR8 (*P*<0.05) (**Supplementary Fig. S5B, C, D**). Post hoc evaluations of GBM patients who survived < 24 months versus those who survived ≥ 24 months demonstrated different GSEA immune-related pathway changes (Fig. 3A; **Supplementary Fig. S6**). The mRNA levels of immune-related genes FER1L5 and TNFSF4 were increased in longer-lived GBM patients at baseline (Fig. 3A). In contrast, genes involved in immune function, motility, protein regulation and signal transduction PTK2, NCKAP1L, BAG6, CRKL, and IL25 were lower in longer-lived GBM patients at the C1 timepoint (Fig. 3B). Additional analysis included comparing younger (<65 years of age) versus older patients (≥65 years of age) at baseline (**Supplementary Fig. S7**) and at the C1 timepoint (**Supplementary Fig. S8**). CD27, which is required for the generation and maintenance of T cell immunity, was noted to be decreased in the older cohort of patients at baseline (**Supplementary Fig. S7**) and the negative regulator of the toll-like receptor IRAK3 was found to be upregulated in older subjects at C1 (**Supplementary Fig. S8**). Different GSEA immune-related pathway changes were also observed when comparing younger (<65 years of age) versus older patients (≥65 years of age) at baseline (**Supplementary Fig. S7**) as well as at the C1 timepoint compared to baseline (**Supplementary Fig. S8**).

Immuno-phenotyping of GBM patient PBMCs using spectral flow cytometry (Fig. 3C, **Supplementary Fig. aS9**) showed differences at baseline and C1 for naïve and early effector CD8^+^ T cells that were increased in GBM patients who survived ≥24 months compared to those who survived <24 months (Fig. 3D). There was a trend for increased levels of activated CD8^+^ T cells in the longer-lived GBM patient group (**Supplementary Fig. S10**). Similarly, patients <65 years of age showed an increased level of early CD8^+^ T effector cells and activated granzyme B^+^ CD8^+^ T cells compared to older counterparts (**Supplementary Fig. S11**). Among the many other types of immune cells analyzed, no other significant differences were found that stratified survival outcomes (**Supplementary Fig. S10, 11, 12**). Since RT contributes to lymphopenia in GBM patients^16^, we investigated absolute lymphocyte count levels during and post RT (Fig. 1A) but did not observe any significant longitudinal changes (**Supplementary Fig. S13**).

To explore additional potential prognostic biomarkers, genomic DNA methylation sequencing of patientderived PBMCs was conducted. Genomic methylation status of two particular CpG sites, cg00553099_BC21 (proximal to the ZNF362 gene that may be involved in transcriptional regulation and associated with other cancers, located on chromosome 1) and cg04256995_BC21 (proximal to SP100 gene – an interferon stimulated nuclear antigen key to innate immune responses, located on chromosome 2), showed a strong ability to prognosticate longer- versus shorter-survivors and were identified based on their maximal hypermethylation and hypomethylation status, respectively (**Supplementary Fig. S14A, B**). To our knowledge, this is the first time these CpG sites have been shown to prognosticate GBM patient survival outcomes. Whether differential methylation can only be useful for predicting outcomes after treatment with RT + PD-1 mAb + IDO1 enzyme inhibitor or whether these CpG sites are also potentially useful for patients treated with other immuno- or SOC-therapies, is unknown. PBMC DNA methylation that varied by host age was distinct from factors associated with stratifying longer- versus shorter-term survival (**Supplementary Fig. S14C**).

### Prolonged survival and intratumoral IDO1 expression

**Figure 4:**
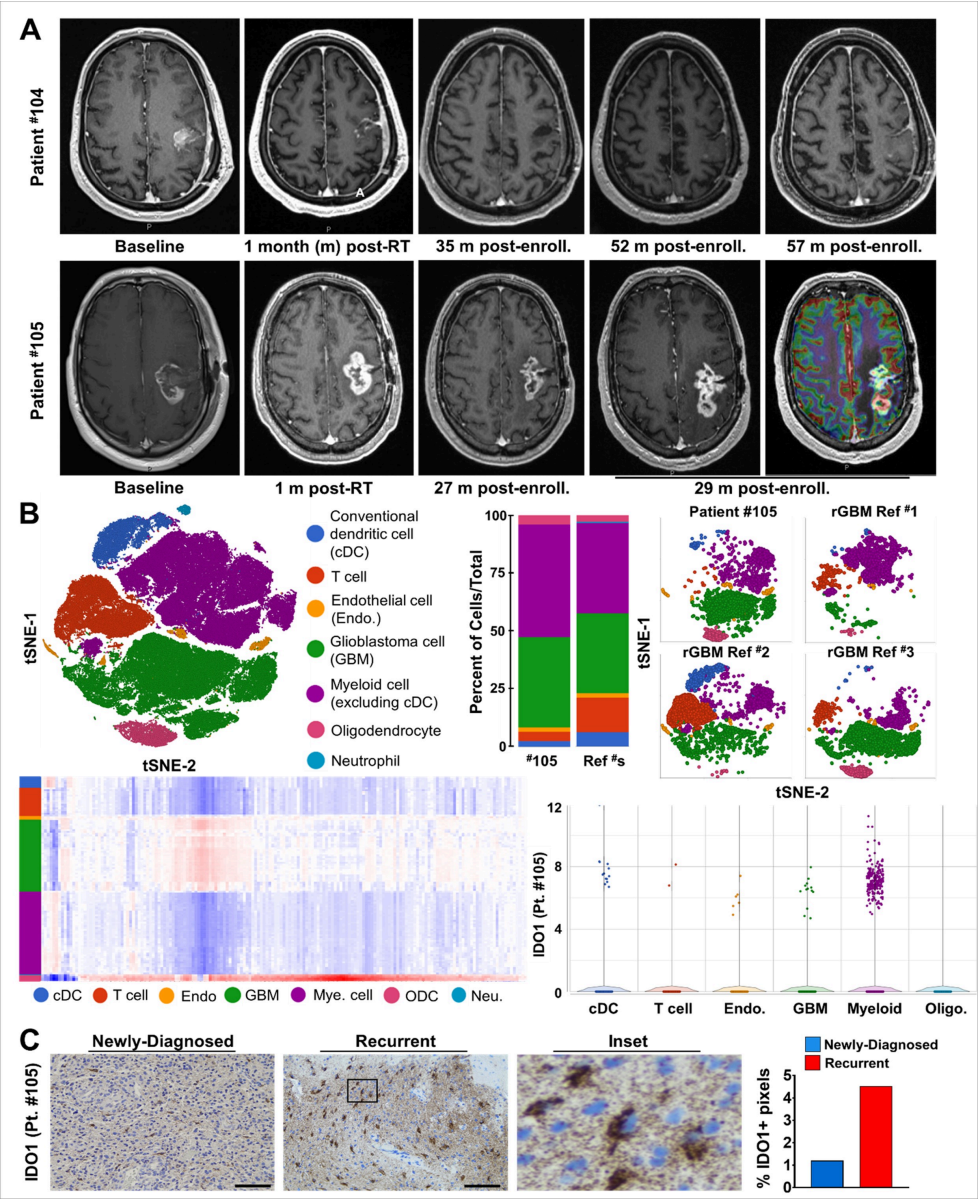
Longitudinal MRI imaging and immune correlatives for long-term survivors in cohort A. **(A)** MRI scans at baseline, one-month post-RT, and more than 29 months after enrollment for patients #105 and for patient #104 that survived >60 months. **(B)** (Left upper quadrant) The combined single-cell RNA-sequencing (scRNA-Seq.) analysis and associated t-distributed stochastic neighbor embedding (t-SNE) plot for different cell types in a rGBM from patient #105, in addition to reference rGBM samples that were previously reported^44^. (Middle upper quadrant) Bar graphs comparing the frequencies for different cell lineages of rGBM from patient #105 or the combination of rGBM reference samples that were previously reported. (Right upper quadrant) A side-by-side comparison of t-SNE plots of rGBM from patient #105 or from 3 separate reference rGBM samples that were previously reported. (Left lower quadrant) A heatmap representing the top 500 differentially expressed genes for each cell lineage in the combined rGBM samples. (Left right quadrant) A violin and box-and-whisker plot representing IDO1 expression across each cell lineage in the rGBM from patient #105. **(C)** Photomicrographs for IDO1 immunoreactivity in GBM tissue specimens resected at the time of initial diagnosis or at tumor recurrence for patient #105. Larger images are displayed in **Supplementary Figure S15**. The inset represents a higher magnification view of IDO1^+^ cells at the time of tumor recurrence. The bar graph represents a quantification of IDO1 immunoreactivity. Scale bar: 100 μm.

Fig. 4A demonstrates the experience of two MGMT promoter unmethylated patients who showed prolonged and sustained radiographic stability at 57 months (patient #104) and 29 months (patient #105). We obtained progressive tumor specimen from patient #105. This tumor was noted to harbor a *BRAF* V600E mutation. Single cell RNA sequencing (scRNA-Seq) investigated the cellular composition of the tumor microenvironment (Fig. 4B) and compared it to previously reported scRNA-Seq data for recurrent GBM samples^17^ (Fig. 4B, single tSNE plot versus multiple tSNE plots) with myeloid-derived cells accounting for the highest proportion of total cells analyzed (Fig. 4B, bar graph of cell type percentage). Gene expression profiling distinguished different intratumoral populations (Fig. 4B, bottom left) with the myeloid compartment representing the largest number of IDO1 expressing cells (Fig. 4B, bottom right). Immunohistochemical staining confirmed an increase of IDO1 immunoreactivity at the time of progression compared to time of initial diagnosis (Fig. 4C; **Supplementary Fig. S15**). IDO1 expression was elevated at the time of tumor progression, but not T cell markers or PD-L1 (**Supplementary Fig. S16**).

### Fecal sample targeted microbiome analysis

**Figure 5:**
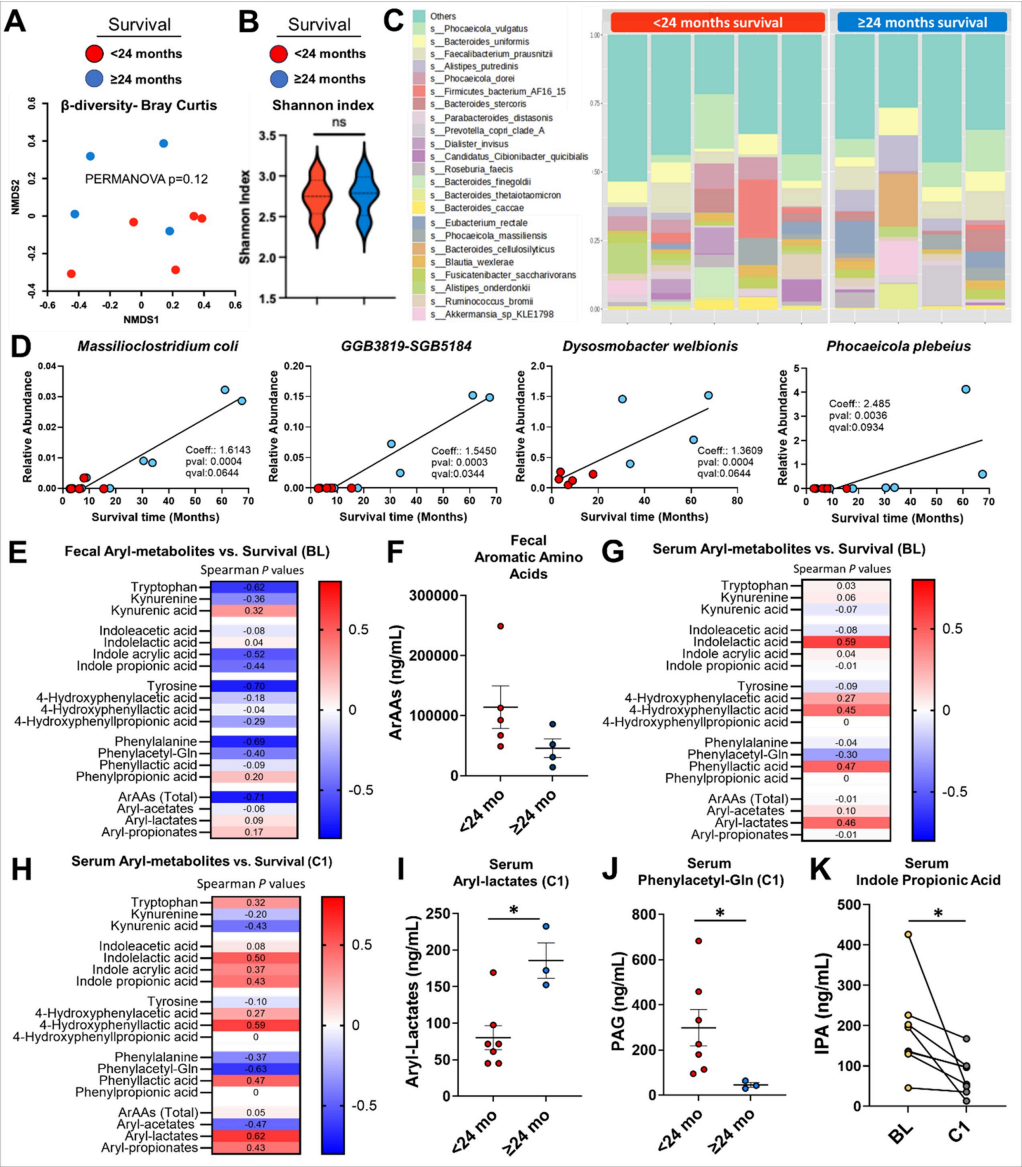
Cohort A microbiome analysis. **(A)**Non-metric multidimensional scaling (NMDS) of the Bray-Curtis index (β-diversity) reveals a trend for gut microbiome composition differences in short- (red; <24 months; n=5) and long-term survivors (blue; ≥24 months; n=4) (PERMANOVA *P*=0.12). **(B)** Alpha-diversity does not differ between short- and long-term survivors. (**C)** Relative abundance of genera (% of total bacteria) in short- and long-term survivors. **(D)** Four microbial species are associated with survival time as assessed through MaAsLin2 (q<0.10). (**E)** Non-parametric Spearman correlations assessing baseline (BL) fecal aromatic amino acids and downstream metabolites vs. future survival time. **(F)** Fecal ArAA levels (Phenylalanine, Tyrosine and Tryptophan) tended to differ between long-term (n=4) and short-term (n=5) survivors (*P*=0.0635). **(G)** Non-parametric Spearman correlations assessing BL serum aromatic amino acids and downstream metabolites vs. future survival time. **(H)**Non-parametric Spearman correlations assessing the first on treatment (C1) timepoint serum aromatic amino acids and downstream metabolites vs. future survival time. **(I)** Aryl-lactates were significantly elevated in long-term (n=3) survivors compared to short-term (n=7) survivors at the C1 timepoint. **(J)** The microbe-host co-metabolite phenylacetylglutamine (PAGln) was lower in long-term (n=3) survivors vs. short-term (n=7) survivors at the C1 timepoint. **(K)** Serum indole-propionic acid (IPA) is depleted in the early treatment phase when comparing BL (n=8) to C1 (n=8). *P<0.05.

Metagenomic sequencing was performed on DNA isolated from fecal samples of GBM patients collected at baseline. β-diversity analysis (Bray-Curtis index) revealed no significant differences in microbial community composition between patients who survived <24 months versus ≥24 months (PERMANOVA *P*=0.12; Fig. 5A). Similarly, no differences in -diversity (Shannon index) were observed between fecal microbiomes of shorter- versus longer-term survivors, suggesting overall microbial richness and evenness were comparable across those different groups (*P*>0.05; Fig. 5B). Fig. 5C illustrates a genus-level comparison of microbial composition across longer- versus shorter-surviving groups with no differences observed. Utilizing MaAsLin2 (Multivariate Association Discovery in Population-scale Meta-omics Studies) analysis^18^, we identified significant correlations between specific microbial species abundance and overall survival. *Massiloclostridium coli (P*<0.0004), *GGB3819-SGB5184 (P*<0.0003; an unclassified species), *Dysomobacter welbionis (P*<0.0004), and *Phocaeicola plebeius (P*<0.0036) were enriched in patients surviving ≥24 months, implicating these taxa as positive indicators and/or effectors of long-term survival in GBM treated with this form of immunotherapy (Fig. 5D; *p* adj<0.10). We also implemented MaAslin2 to correlate functional microbiota pathways with survival. Notably, pathways associated with *Bacteroides plebeius* including rhamnose degradation, positively correlated with long-term survival, whereas pathways linked to *Bacteroides xylanisolvense* associated with coenzyme A biosynthesis essential to multiple metabolic pathways, and *Coprococcus catus* associated with aminoimidazole biosynthesis, negatively correlated with survival (*P* adj<0.05; **Supplementary Fig. S17A**). Aminoimidazole biosynthesis plays a role in the *de novo* purine synthesis pathway that’s associated with glioblastoma progression^19^. We compared patients who survived more than 60 months to those with shorter survival times <60 months and discovered five bacterial taxa including *Acidaminococcus intestini*, *Phocaeicola plebeius*, *Allisonella histaminiformans*, *Massiloclostridium coli*, and *Barnesiella intestinihominis*, that were enriched in the longer- versus shorter-surviving GBM patients (**Supplementary Fig. S17B**).

### Host- and microbial-derived metabolite analysis

Although our primary goal was intended to inhibit IDO1 enzyme activity in the tumor, it is well established that IDO1 is also highly expressed along the gastrointestinal tract by gut epithelial cells and select types of immune cells^20^. We hypothesized that IDO1 enzyme inhibitor treatment would elicit direct effects on gut microbiota metabolism. Given similarities to indole-based kynurenines, we focused our analysis on microbial-derived aromatic amino acid (ArAA) metabolites. The primary difference between aryl metabolites versus aromatic amino acids is that the latter contains carboxyl (-COOH) function groups attached to an aromatic system. Using high-sensitivity targeted liquid chromatography with tandem maurss spectrometry (LC/MS/MS), we assessed fecal aryl-metabolites at baseline in relation to patient survival (Fig. 5E). While no associations were observed between fecal microbial-derived aryl-metabolites and survival (*P*>0.05), fecal aromatic amino acids (Phe, Tyr, and Trp) showed a significant negative correlation (*P*<0.05; Fig. 5E). Fecal ArAAs strongly trended toward differences between long- and short-term survivors (*P*=0.06; Fig. 5F). We further analyzed the plasma-derived aryl-metabolites and found significant associations between microbial-derived aryl-lactates, particularly indole lactic acid (ILA) and survival (*P*<0.05; Fig. 5G). At C1, ILA along with other aryl-lactates including phenyllactic acid and 4-hydroxyphenyllactic acid, positively correlated with survival (*P*<0.05; Fig. 5H). Consistent with this, aryl-lactates were significantly elevated in long-term compared to short-term survivors (Fig. 5I; *P*<0.05). In contrast, microbe-host co-metabolite phenylacetylglutamine (PAG) showed a negative correlation with survival (*P*<0.05; Fig. 5H) and was lower in long-term survivors (*P*<0.05; Fig. 5J). Additionally, a subset of patients with both baseline and C1 serum samples demonstrated depletion of indole-propionic acid (IPA) (*P*<0.05; Fig. 5K).

## DISCUSSION

In this phase 1 and translational study we demonstrated that the anti-PD1 monoclonal antibody nivolumab in combination with an oral IDO inhibitor (BMS-986205) can be safely combined with standard of care radiotherapy and chemotherapy for newly diagnosed GBM. While the mOS was in the expected range for this disease, one third of the patients with MGMT unmethylated tumor demonstrated prolonged survival beyond 2 years. This compares favorably to the ~10% of MGMT unmethylated GBM patients who were treated with dual RT + nivolumab and survived beyond 24 months as reported in the Checkmate 498 trial – especially as the latter study also included mutated IDH-1 patients^21^. One of the longest survivors possessed a somatic BRAF mutation in his initial tumor sample (Patient Clinical Data in **Data Source**). GBM IDHwt with BRAF mutations and other MAPK pathway aberrancies have been shown to be associated with superior outcomes after treatment with PD-1 mAb immunotherapy^22,23^. Notably, patients who underwent an incomplete resection experienced better outcomes than patients who underwent a GTR. A similar finding has been reported in multiple phase 3 clinical trials, as well as during neoadjuvant immune checkpoint blockade^24–27^.

We demonstrated that although BMS-986205 significantly reduces serum *L*-kynurenine and 3-hydroxykynurenine levels in GBM patients, the small magnitude of those changes raises questions about their biological significance. Our study was not designed to answer questions regarding the impact of kynurenines within the TME. Despite the potency of BMS-986205 with an IC_50_ of 1.7nM, no changes were observed in serum *L*-tryptophan in treated GBM patients, highlighting the robust homeostatic mechanisms that maintain normal systemic levels of this essential amino acid. We failed to find significant changes for other downstream kynurenine-pathway metabolic products in the serum including kynurenic acid, picolinic acid, quinolinic acid, anthranilic acid, or 3-hydroxy-anthranilic acid that, purportedly depend on *L*-kynurenine as an upstream resource for their own generation. Serum *L-*kynurenine levels were also not different between long-term survivors and short-term survivors, suggesting that any potential benefits of the IDO1 enzyme inhibitor may be independent of its enzymatic activity *or* that serum levels are a poor proxy of CNS and specifically intra-tumoral levels. Given that small amounts of IDO1 are extracellularly released^28^, and since extracellular IDO1 induces immunosuppression in myeloid lineage cells^29^, it’s possible that IDO1 enzyme inhibitor treatment non-enzymatically changes the function(s) of extracellularly-released IDO1. This hypothesis is in-line with our previous finding that tumor cell IDO1 non-enzymatically decreases survival in a preclinical brain tumor model but the mechanism associated with that effect has yet to be elucidated^30^.

At the time of tumor recurrence, intra-GBM IDO1 RNA expression was primarily restricted to the myeloid cell lineage, and predominantly, microglia. We are mindful that the patient whose sample was analyzed had an initial prolonged interval of radiographic stability and that our tissue analysis was performed at the time point where treatment failed to prevent tumor progression. There are a number of possibilities for the qualitatively increased IDO1 protein expression and cellular localization at the time of tumor recurrence. Since the induction or upregulation of IDO1 is exquisitely sensitive to inflammatory stimuli^8,11^, the increased IDO1 expression may simply reflect enhanced levels of inflammatory drivers in the recurrent tumor microenvironment. With the notable expression in myeloid derived immune cells, this active negative feedback response to inflammation may represent a key mechanism fostering the immunosuppressive TME in progressive GBM. Another possibility is that the IDO1 enzyme inhibitor treatment caused a reciprocal compensatory accumulation of IDO1 protein expression attempting to maintain homeostasis of this key immunomodulatory nidus. This IDO1 accumulation is consistent with previous reports^31,32^.

Despite the prolonged survival for several patients, it is important to acknowledge that IDO1 enzyme inhibitor treatment has failed to produce survival improvements in several randomized phase 3 non-CNS tumor trials and early phase glioma trials to-date^33–37^. However, those studies did not incorporate IDO1 inhibitor with concurrent radiotherapy. The RT component of our therapeutic approach is presumably a critical factor that initiates an inflammatory cascade accompanied by an interferon driven induction and activation of IDO1^11^. As we previously reported in a preclinical study, the IDO1 enzyme inhibitor synergizes to improve long-term survival only when combined with RT and PD-1 mAb – and not dual- or mono-therapeutic combinations^13^.

Building on the potential non-enzymatic effects of the IDO1 enzyme inhibitor, we next examined the role of the microbiome, particularly its influence on aromatic amino acid metabolism and its relationship to GBM patient survival outcomes in the context of immunotherapeutic treatment. The microbiome has well-established functional consequences on the efficacy of cancer immunotherapy^38^, including for glioma^39^. Recent investigations are beginning to clarification on how microbiome signatures can help stratify beneficial GBM patient outcomes after treatment with immunotherapy^40^. Our investigation into microbial metabolite levels in the blood and metagenomic microbial signatures in the stool of GBM patients was driven by the hypothesis that disruptions in the gut↔brain axis affect GBM patient outcomes after treatment with RT, nivolumab, and BMS-986205.

Notably, IDO1 is constitutively and highly expressed along the gastrointestinal (GI) tract in immune and epithelial cells, making it a critical source of interaction between the microbiome and the host.^41^ Consistent with the preclinical findings^42,38^, we observed significantly higher levels of microbial-derived aryl-lactates and lower levels of phenylacetylglutamine (PAG) in the blood of patients who survived more than 24 months in our trial. Furthermore, patient survival was strongly and positively correlated with increased fecal abundance of *Massilioclostridium coli*, *Dysosmobacter welbionis*, and *Phocaeicola plebeius*. Interestingly, several microbes including *Acidaminococcus intestini*, *Phocaeicola plebeius*, and *Allisonella histaminiformans*, were exclusively detected in the stool of GBM patients who survived ≥5 years. That distinct microbial species and aryl-metabolites are predictive of immunotherapy cancer survival align with recent studies, with evidence that both PAG and *Allisonella histaminiformans* are associated with pan-cancer survival responses to immunotherapy^43^. Our group is actively investigating how age and IDO1 interact within the gastrointestinal tract and how these interactions potentially influence the gut microbiota and microbial metabolites in GBM patients treated with immunotherapy. This clinical trial warrants further investigation into the underlying mechanisms of action, treatment resistance, and predictive biomarkers associated with patients treated with concurrent RT, nivolumab, and IDO1 enzyme inhibition.

## Supplementary Files

This is a list of supplementary files associated with this preprint. Click to download.

- DataSource.xlsx
- SUPPLEMENTARYTABLESFIGURES.docx

## Figures and Tables

**Table 1:** Demographic and baseline clinical characteristics of patients with MGMT promoter unmethylated GBM (Cohort A) and MGMT promoter methylated GBM (Cohort B).

Characteristics	Cohort A			Cohort B
	Overall, N = 12	BMS-986205 Dose Arms		Overall, N = 6
		50mg, N = 6	100mg, N = 6	
**Age (years) at registration**				
Median (IQR)	58.0 (49.8, 66.5)	49.5 (46.8, 56.8)	63.5 (58.0, 68.3)	64.5 (60.0, 70.5)
Range	41.0, 65.0	41.0, 66.0	50.0, 69.0	55.0, 72.0
**Age stratification at 55-y**				
< 55	5 (41.7%)	4 (66.7%)	1 (16.7%)	
>= 55	7 (58.3%)	2 (33.3%)	5 (83.3%)	6 (100%)
**Age stratification at 65-y**				
<65	8 (66.7%)	5 (83.3%)	3 (50.0%)	3 (50.0%)
>= 65	4 (33.3%)	1 (16.7%)	3 (50.0%)	3 (50.0%)
**Sex**				
Female	5 (41.7%)	2 (33.3%)	3 (50.0%)	2 (33.3%)
Male	7 (58.3%)	4 (66.7%)	3 (50.0%)	4 (66.7%)
**Race**				
Unknown	2 (16.7%)	2 (33.3%)	0	0
White	10 (83.3%)	4 (66.7%)	6 (100.0%)	6 (100%)
**Ethnicity**				
Hispanic or Latina	1 (8.3%)	0 (0.0%)	1 (16.7%)	0
Non-Hispanic	10 (83.3%)	5 (83.3%)	5 (83.3%)	6 (100%)
Unknown	1 (8.3%)	1 (16.7%)	0	0
**KPS**				
70–80	5 (41.7%)	2 (33.3%)	3 (50.0%)	1 (16.67%)
90–100	7 (58.3%)	4 (66.7%)	3 (50.0%)	5 (83.33%)
**Tumor-treated fields (TTF)**				
Yes	4 (33.3%)	1 (16.7%)	3 (50.0%)	4* (66.7%)
No	8 (66.7%)	5 (83.3%)	3 (50.0%)	2 (33.3%)
**MGMT promoter status**				
Unmethylated	12 (100.0%)	6 (100.0%)	6 (100.0%)	0
Methylated	0	0	0	6 (100%)

*: one patient started TTF after the end of treatment (EOT)

IQR: interquartile range; KPS: Karnofsky Performance Status

## Data Availability

All raw sequencing data included in this manuscript is either available at the GEO deposition sources indicated in the materials and methods section, or is available upon request from the corresponding authors.
